# Haem iron versus ferrous iron salts to treat iron deficiency anaemia in Gambian children: protocol for randomised controlled trial {1}

**DOI:** 10.1186/s13063-024-08101-0

**Published:** 2024-04-19

**Authors:** Mamadou Bah, Hans Verhoef, Emmanuel Okoh, Abdoulie Bah, Andrew M. Prentice, Carla Cerami

**Affiliations:** 1 Medical Research Council (MRC) Unit The Gambia at London School of Hygiene & Tropical Medicine (LSHTM), PO Box 273, Fajara Banjul, The Gambia; 2 Division of Human Nutrition and Health, Wageningen University, PO Box 17, 6700 AA Wageningen, The Netherlands

**Keywords:** Iron deficiency, Anaemia, Iron supplements, Haem iron, Gambia, Infants, Iron status, Safety, Hepcidin

## Abstract

**Background:**

The World Health Organization recommends universal iron supplementation for children aged 6–23 months in countries where anaemia is seen in over 40% of the population. Conventional ferrous salts have low efficacy due to low oral absorption in children with inflammation. Haem iron is more bioavailable, and its absorption may not be decreased by inflammation. This study aims to compare daily supplementation with haem iron versus ferrous sulphate on haemoglobin concentration and serum ferritin concentration after 12 weeks of supplementation.

**Methods:**

This will be a two-arm, randomised controlled trial. Gambian children aged 6–12 months with anaemia will be recruited within a predefined geographical area and recruited by trained field workers. Eligible participants will be individually randomised using a 1:1 ratio within permuted blocks to daily supplementation for 12 weeks with either 10.0 mg of elemental iron as haem or ferrous sulphate. Safety outcomes such as diarrhoea and infection-related adverse events will be assessed daily by the clinical team (see Bah et al. Additional file 4_Adverse event eCRF). Linear regression will be used to analyse continuous outcomes, with log transformation to normalise residuals as needed. Binary outcomes will be analysed by binomial regression or logistic regression, Primary analysis will be by modified intention-to-treat (i.e., those randomised and who ingested at least one supplement dose of iron), with multiple imputations to replace missing data. Effect estimates will be adjusted for baseline covariates (C-reactive protein, alpha-1-acid glycoprotein, haemoglobin, ferritin, soluble transferrin receptor).

**Discussion:**

This study will determine if therapeutic supplementation with haem iron is more efficacious than with conventional ferrous sulphate in enhancing haemoglobin and ferritin concentrations in anaemic children aged 6–12 months.

**Trial registration:**

Pan African Clinical Trial Registry PACTR202210523178727

**Supplementary Information:**

The online version contains supplementary material available at 10.1186/s13063-024-08101-0.

## Background {6a and 6B}

In sub-Saharan Africa, conventional iron supplementation using ferrous salts has limited effectiveness, primarily due to impaired non-heme iron absorption caused by infections and inflammation. Although haem iron has greater bioavailability, it has never been evaluated in children in an LMIC setting. In a before-after study without a control group among pregnant Kuwaiti women with gestational age 14–26 weeks with an initial haemoglobin concentration of 80–10 g/L, daily supplementation with haem iron tablets (Optifer®; 1 tablet twice/day) for ≥ 3 months resulted in notable increase in the mean haemoglobin concentration (from 84 to 112 g/L; *p* < 0.003) and mean ferritin concentrations (from 22.6 to 112.8 μg/L; *p* < 0.04). Only 1.7% (2/117) of the women studied developed gastrointestinal adverse effects, and no other side effects were recorded [1]. The present trial will compare the supplementation with 10 mg of haem iron versus ferrous salts in young Gambian children (ages 6–12 months) using a commercially available haem iron polypeptide (HIP) produced by hydrolysis of bovine haemoglobin.

Most iron supplementation trials, and all institutional programmes in Africa, use ferrous iron salts (typically sulphate or fumarate). These are cheap but have low efficacy, particularly in children living in rural Africa [2]. The very low efficacy of iron administration is a universal problem across all iron supplementation trials in low- and middle-income countries [2]. This is likely driven, at least in part, by hepcidin-mediated blockade of iron absorption secondary to infections and inflammation [3]. As an example, we have recently completed 2 trials in rural Gambia with 400 children aged 6–24 months [4] and 500 second trimester pregnant women [5]. Despite administering iron for 12 weeks (12 mg/day to children and 60 mg/day to pregnant women) with an additional 15 micronutrients and with all doses taken under direct supervision, we only achieved a 0.65 g/dL increase in haemoglobin concentration in the children and 0.33 g/dL in the mothers. The prevalence of anaemia dropped only from 91 to 77% and from 59 to 45%, respectively.

Haem iron is the most bioavailable form of iron and is readily available from meat, poultry, and some types of fish [6, 7]. Haem iron uptake through enterocytes is incompletely understood in children, but it is known to travel, at least in part, via an alternative absorption pathway to non-haem iron. This hypothesis is supported by the following evidence: (1) there was no evidence that hepcidin concentrations affected haem iron absorption in a study of pregnant (*n* = 18) and non-pregnant (*n* = 11) women [8], and (2) once inside enterocytes, haem induces the degradation of the transcriptional repressor Bach1 [9]. Bach1 regulates haemoxygenase-1, so that haem manages to ensure its own detoxification by increasing levels of HO-1, which liberates iron from haem. However, Bach1 also represses ferroportin [10]. Thus, haem uptake both facilitates the release of iron from the porphyrin ring within the enterocyte and its subsequent release from the enterocyte into the circulation via ferroportin. The strong upregulation of ferroportin at the transcriptional level, mediated by haem-induced Bach1 degradation, could potentially counteract the inhibition of ferroportin protein by hepcidin.

## Objectives {7}

The primary objective will be to determine if the efficacy of haem iron supplementation using a commercially available haem iron polypeptide (HIP) is superior compared to conventional ferrous sulphate.

## Methods/design {8}

### Study design

This will be a 2-arm parallel randomised controlled superiority trial, with participants, field staff outcome assessors and biostatisticians blinded to the treatment arm. The trial will be conducted in rural Gambia, and potential participants will be recruited from vaccination clinics or their communities. A total of 208 participants will be randomised to either haem iron or ferrous sulphate using a 1:1 allocation ratio and supplemented daily for 84 days (Fig. 2).

### Ethics and oversight {5d, 23, 24 and 25}

The study was approved by the Scientific Coordinating Committee of the Medical Research Council (MRC) Unit The Gambia and the Joint Gambia Government MRC Ethics Committee and the Ethics Committee at the London School of Hygiene and Tropical Medicine (LEO27648). Protocol amendments will be submitted through the same committees. This trial will be conducted according to the principles of Good Clinical Practice, with oversight from a Data Safety and Monitoring Board (DSMB). The Clinical Trials Department at the MRCG@LSHTM (dct@mrc.gm) will audit the trial. The Clinical Trials Department will conduct a site initiation visit followed by regular visits. The first interim monitoring visit (IMV) will be done once signed informed consent is obtained from at least 50 participants. Thereafter, a visit will be done monthly. The frequency of the interim visits may be scaled down or increased if required at the discretion of the sponsor.

#### Composition of the data monitoring board, its role and reporting structure {21a}

An independent data safety management board (DSMB) was constituted before the start of the study to provide oversight. It is comprised of four members including two area experts, one clinical research specialist and one trial statistician. The DSMB will be responsible for reviewing safety data and data related to recruitment and retention. The role of the DSMB will be to advise the sponsor and trial management group if, in their view:

- There is a level of concern about the safety of participants in any treatment arm such that future recruitment to that arm is no longer judged to be appropriate.
- The quality of the safety data generated is of such concern (in terms of completeness and accuracy) that an assessment of the participants’ safety is not considered to be possible.
- The quality of the safety and other data generated is of such significant concern (in terms of completeness or accuracy) that the results of the trial are likely to be called into question irrespective of the outcome.
- The rate of recruitment is such that it is unlikely that the trial will be completed successfully.
- They become aware of other data addressing the same or related questions to those in the IDeA3 study such that the trial is no longer considered to be justified in its current form.

The charter of the DSMB is kept in the Trial Master File and is available on request.

#### Trial sponsor {5b and c}

The trial sponsor is the Medical Research Council Unit The Gambia at London School of Hygiene & Tropical Medicine, P.O. Box 273, Banjul, The Gambia, West Africa. Neither the funders nor the sponsor will be involved in the conduct of the study.

#### Confidentiality {27}

To safeguard the confidentiality of participants, all identifiable data collected by the study team will be securely stored and protected in accordance with the Data Protection Act. If there is a need to share data about participants through a public data repository or directly with other researchers, precautions will be implemented to ensure that participant information remains unidentifiable. All data sharing will adhere to the guidelines outlined in the UK/European General Data Protection Regulation (GDPR).

### Participant retention {18b} and post-trial care {30}

Participants will be able to withdraw from the study at any time without providing a reason for their withdrawal. However, if participants provide a reason, this will be documented with the date of participant discontinuation. If an adverse event is associated with the participant’s discontinuation, it will be described, even if it is not the primary reason for withdrawal.

All participants will be passively monitored by the clinical team during the study and 2 weeks post-supplementation for any adverse (AE) or serious adverse events (SAE).

### Recruitment and screening {9, 11c, 15 and 18b}

Before the study begins, meetings with local elders and community leaders and group meetings with community members in the villages within the recruitment area (see Additional file 1: Table S1: list of villages that will be included) will be conducted to inform and educate the general public and potential participants about the study. To ensure effective communication and integration in the communities, field workers will be assigned to participants based on their language fluency (e.g. Mandika, Fula, Wolof). Potential participants will be identified in vaccination clinics or in their homes by trained field workers who will explain the study to the parents or guardians individually.

### Who will take informed consent? {26a, b}

The discussions about the trial and informed consent will take place in a private location chosen by the participants. For illiterate participants, an impartial witness will be present. Before participants provide informed consent, the field staff will assess their understanding using an electronic case report form (eCRF). If participants fail the assessment, the study information will be explained to them again. If participants fail the assessment for a third time, consent will not be obtained, and this will be documented as consent failure in the relevant eCRFs. Participants who refuse to provide consent or fail the screening will not be enrolled in the study, and this will be recorded in the relevant eCRFs. Anaemic participants will be invited for a baseline screening and eligible individuals enrolled in the study. Note that the informed consent document contains the following sentence: “I agree to further research on my child’s samples including genetic testing.”

**Fig. 1:**
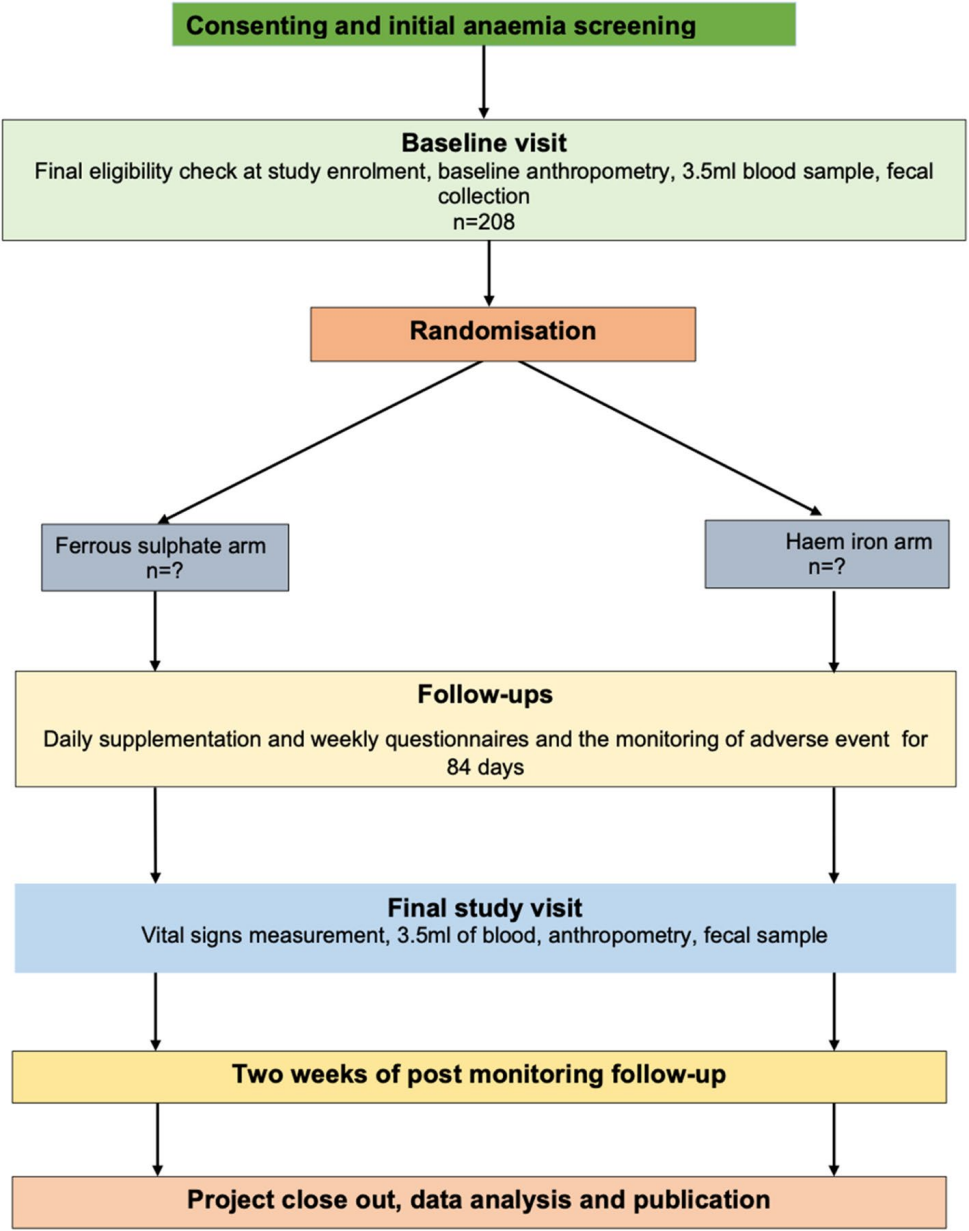
Schematic of the study design

The follow-up period will be 84 days (12 weeks) with 2 weeks of post-study follow-up for adverse events (Fig. 1). Figure 1 shows a schematic of the study design {13}. Table 1 shows the sequence of events in the trial from the perspective of the study participants (Table 1).

**Table 1:** Sequence of events

Study activities	Prior to enrolment	Intervention period												Follow-up	
	Week (Wk) 0	Wk 1	Wk 2	Wk 3	Wk 4	Wk 5	Wk 6	Wk 7	Wk 8	Wk 9	Wk 10	Wk 11	Wk 12	Wk 13	Wk 14
**Informed consent**	x														
**Pre-screening for anaemia/eligibility**	x														
**Demographic data collection**		x													
**Anthropometry measurement**		x											x		
**Screening for inclusion and exclusion**		x													
**Baseline sample collection**		x													
**Randomisation**		x													
**Daily supplementation**		x	x	x	x	x	x	x	x	x	x	x	x		
**Weekly feeding questionnaire**		x	x	x	x	x	x	x	x	x	x	x	x		
**Monitoring of adverse events**		x	x	x	x	x	x	x	x	x	x	x	x		
**Endline sample collection**													x		
**Post supplementation follow-up**														x	x

### Eligibility criteria {10}

Once consent is provided by parents/guardians, participants will be screened for anaemia (Hb < 11.0 g/dL) using HaemoCue 301 (HemoCue®, Ängelholm, Sweden). Participants who are anaemic will be invited to the study sites for further eligibility screening through the assessment of the inclusion and exclusion criteria by study nurses or research clinicians. We will include children between the ages of 6 to 12 months of age who are anaemic (Hb 7– <11.0 g/dL). Any children who are acute or chronically ill, have Hb < 7.0 g/dL, have fever (axillary temperature > 37.5 °C), history of low birth weight (< 1500 g), prematurity (born gestational age < 34 weeks), are planning to use of vitamin supplements or have any condition that in the opinion of the investigator or clinical team might compromise the safety or well-being of the participant or compromise adherence to protocol procedures will be excluded. Children who are found to be unwell during the screening process will be referred for care to a local clinic.

### Randomisation and blinding to allocation and treatment {16a, b and c and 17a}

Prior to baseline sample collection, a list of participant IDs will be generated. The participant ID will be comprised of a four-letter study code (IDEA), a four-digit unique number (e.g. 0001–3000) and a check letter (e.g. A–Z). Once participant eligibility is confirmed, the sub-investigator or data manager will assign each participant a unique intervention code (i.e. 1–6) by running the electronic randomisation CRF in RedCap. The password-protected randomisation eCRF will be developed and will only be accessible by the data manager. In order to blind the treatments, there will be six intervention codes (1–6) of which three will be assigned to the ferrous sulphate arm and the other three to the haem iron arm by the pharmacist (see the section describing IP below). The treatment randomisation code will be kept in a locked box, only accessed if unblinding becomes necessary. The study IDs with their respective treatment codes will be exported from RedCap as a PDF and sent to the clinical team who will issue the assigned treatment to the field team daily. To maintain the integrity of blinding, the randomisation code will be stored in a sealed, time-stamped envelope. This envelope will be securely kept in a locked box and will only be accessed if unblinding becomes necessary. The data collectors, participants, investigators and biostatisticians will be blinded to the intervention.

### Intervention {11a}

The haem iron will be purchased commercially packaged as a black powder in clear capsules (Proferrin, Colorado Biolabs, USA). Ferrous sulphate iron will be purchased commercially as a black powder in a green capsule (Active Iron, Solvotrin, Ireland). A pharmacist will repackage both investigational products into identical clear capsules containing 10 mg of each product to ensure blinding. The intervention products will be divided into six intervention arms, labelled as arms 1 to 6. The pharmacist will label the bags containing the capsules with the investigational products (IP) based on the randomisation codes. As described above, the study team and participants will be blinded to the type of intervention given.

The intervention products will be stored at the field office, where the temperature will be monitored daily. The field staff will transport all intervention products to participants’ homes daily for 84 days. They will open the capsules and mix the powder with water in a teaspoon with the assistance of the parent/guardian to administer it to the children. If a participant vomits or spits out the intervention, a second dose will be re-administered and this information will be recorded.

### Procedure for unblinding if needed {17b}

Unblinding of the treatment will only be initiated by the Data Safety Monitoring Board (DSMB) on an individual basis in the event of a consistent occurrence of serious adverse events potentially related to the study treatment, raising concerns about potential harm. Causality will be determined through discussions between the principal investigators (PIs), the study clinician with input from the local safety monitor (LSM). Throughout the unblinding process, the interpretation of the intervention codes will not be documented in any paperwork accessible to the blinded team unless absolutely necessary. If documentation in accessible paperwork is unavoidable, the assumption of complete unblinding for that participant will be made.

### Relevant concomitant care permitted or prohibited during the trial {11d}

Participants who are planning to use any vitamin supplements during the trial will be excluded from enrolment. Once enrolled, participants will only be prescribed with vitamins by the research clinician when deemed necessary.

### Assessments {18a}

#### Laboratory and clinical evaluations {33}

At both baseline (day 0) and at the endline (day 84), 0.5 ml and 3.0 ml of venous blood will be collected into EDTA (BD Microtainer, K2E, REF; 365975, USA) and serum separator tubes (BD vacutainer, REF;367957, UK). Parents/guardians will be provided with baby diapers and instructed to use them to collect stool in the mornings before the baseline and endline visits. Blood samples will be processed within 4 h after collection by trained lab personnel in a GCLP-accredited laboratory. The EDTA samples will be used to assess the full blood count and reticulocyte markers using an automated haematology analyser (Sysmex XN-Series 1500, Sysmex Corporation, Japan). The serum samples will be stored at − 80 °C until subsequent analysis of iron status markers (ferritin, transferrin, transferrin saturation, unbound iron-binding capacity, soluble transferrin receptor) and inflammatory markers [C-reactive protein (CRP) and α-1-acid glycoprotein (AGP)] using a Cobas Integra 400 plus (Roche Diagnostics, Rotkreuz, Switzerland). ELISAs will be used to measure serum concentrations of hepcidin (DX-EIA-5782, DRG, Germany), erythropoietin (DX-EIA-3646, DRG, Germany) and erythroferrone (ERF-001, Intrinsic Lifescience LLC, USA).

Faecal samples from the diapers will be collected and placed into a container and placed on ice. The samples will be transported to the Keneba Laboratory. Stool will then be assessed for calprotectin (MRP 8/14, DX-EIA-5415, DRG, Germany), myeloperoxidase (in-house ELISAs) and gut pathogens (in-house DNA analysis).

Additionally, anthropometric measurements (weight and height at baseline and endline), vital signs, adverse events and qualitative data such as screening, demography, daily iron supplement and weekly feeding questionnaires will be collected. All laboratory analysis results will be captured electronically in the REDCap project database. The blood and faecal samples collected during the trial may be used to support other research in the future and may be shared anonymously with other researchers, for their ethically approved projects. Any future use of data or samples will require approvals from the principal investigator, MRCG@LSHTM Scientific Coordinating Committee, and Ethics Committee.

### Adverse event assessment and reporting {11b}

All adverse events and serious adverse events will be recorded by the research clinician or nurses in RedCap and treatment/referral provided where required. Bi-weekly meetings with the field team will be held and a summary of field activities including the number of participants who consented, screened and any adverse events reported. Field workers will immediately call the research clinician/study nurse if they find a participant is unwell and will seek advice if the investigational product should be withheld. Reported fever will be confirmed by measuring the axillary temperature using a digital thermometer in the field by field workers. The outcome will be reported to the clinical team immediately who will decide on treatment/clinical referral where required. All symptoms or signs reported or observed will be documented after evaluation by the study nurse or clinician.

Investigational products shall be withheld for 7 days following the detection of the following conditions: (1) confirmed fever (axillary temperature > 37.5 °C) not associated with teething or vaccination, (2) visually confirmed bloody diarrhoea, (3) hospitalisation for somatic illness and (4) treatment with antibiotics for any confirmed or suspected infection.

#### Adverse event reporting and harms {22}

Adverse events whether related or unrelated to the interventions will be captured. These will include respiratory tract infections (including upper respiratory tract infections, pneumonia and bronchiolitis), gastrointestinal (gastroenteritis, acute watery diarrhoea, persistent diarrhoea and bacillary dysentery) and skin infections (skin infection, abscess and conjunctivitis) (Additional file 4_Adverse Event eCRF). Diarrhoea will be defined as three or more fluid stools as reported by parents/guardians within the previous 24-h recall period [11]. Acute watery diarrhoea will be defined as diarrhoea that lasts several hours or days [11]. Persistent diarrhoea will be defined as diarrhoea that lasts 14 days and longer with three of more days between episodes [11].

### Data management {19}

Only those individuals who have received training on the CRF/eCRFs and/or other document completion and have been authorised by the principal investigator will complete CRFs and other study-specific documentation. The study data collected on the eCRFs will be captured using devices, i.e. tablets and/or laptops, used by the study staff. Quality control checks will be conducted by the data manager (DM) as data is received. Any missing data identified will result in queries being assigned to the respective data collectors for correction. An internal monitor will oversee the study to ensure data quality and compliance with the International Council for Harmonisation of Technical Requirements for Pharmaceuticals for Human Use (ICH GCP) standards.

The database locking process will be initiated when the study reaches its predefined closure time. The database will be thoroughly reviewed, cleaned and checked by the data manager and their team. Once the database is ready for locking, user access will be removed, and a backup copy will be sent to the Archive Department. The data manager and statistician will be responsible for executing the database locking process, ensuring data completeness and accuracy before statistical analysis. The final data format can be exported to Excel and commonly used statistical packages such as SPSS, SAS, Stata and R.

### Outcome measures {12}

The primary and secondary outcomes will be collected at baseline (day 0) and endline (day 84) at the study sites. The *primary endpoints* are haemoglobin concentration and serum ferritin concentration at day 84. Secondary endpoints related to the primary objective will include proportion of children with anaemia (haemoglobin concentration < 11.0 g/dL) [12] at study day 84, proportion of children with iron deficiency (ID) (ferritin < 12 μg/L or < 30 μg/L in the presence of inflammation) [13] and proportion of children with iron deficiency anaemia (IDA) (haemoglobin concentration < 11.0g/dL and sTfR mg/L/logFerritin ratio > 2.0, ferritin < 30 μg/L in the presence of inflammation (CRP > 5 mg/L)) [14] at day 84. We will also determine other haematology (MCV, MCH, MCHC, reticulocyte-haemoglobin concentration and reticulocyte count) and iron status markers (iron, transferrin, soluble transferrin receptor, hepcidin, erythropoietin, erythroferrone, transferrin saturation, unbound iron binding capacity) at day 84. We will also measure the effect of the intervention on growth.

Safety measures will consist of the incidence of adverse events and serious adverse events per child over the observation period and serum concentrations of inflammatory markers (C-reactive protein/α1-acid glycoprotein) at day 84 and faecal calprotectin.

### Statistical plan

#### Sample size determination {14}

**Fig. 2:**
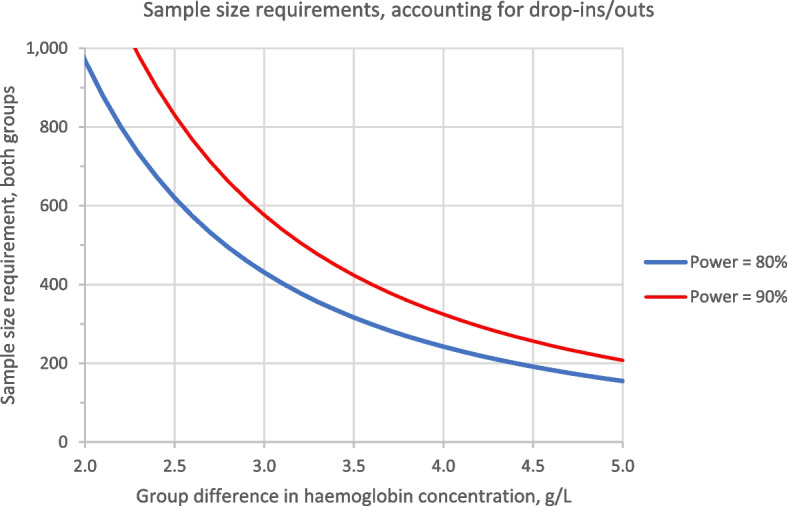
Sample size requirements for various intervention effect size

The sample size was determined by the primary endpoints, haemoglobin concentration and serum ferritin concentration, which will both be measured 84 days after randomisation (Fig. 2) using Microsoft Excel™ with a 5% dropout rate; a total of 208 children will be randomised, which will be sufficient to (a) provide 90% probability that, if the real group difference in haemoglobin concentration is 5.0 g/L or more, the 95% CI for the difference would exclude 0 [calculation based on the assumption that the SD for haemoglobin is 10g/L for each group (estimate based on previous experience in many previous trials in African children)] and (b) have 90% probability that, if the real change of the geometric mean serum ferritin concentration in the haem group iron relative to the control group is 75% or more, the 95% CI for the ratio would exclude 0 [calculation based on the additional assumption that the variance of log_e_-(serum ferritin concentration, μg/L) equals 1.232 for each group [15]].

In absolute terms, an effect of 75% change in serum ferritin concentration would correspond to an increase in the geometric mean value from 13.4 μg/L [estimate obtained from Gambian children with similar age range in a previous study [15]] to 23.4 μg/L (difference, 10 μg/L).

#### Statistical analysis {20a}

Statistical analysis will be conducted using the R Studio (R-4.3.3; https://cran.r-project.org/) and STATA software (vs 18; StataCorp, College Station, TX, USA). The intervention groups will be characterised at baseline and end survey using conventional summary statistics such as prevalence, means or counts with standard deviations (SDs), geometric means (geometric SDs) or medians with 25th and 75th centiles, as appropriate. We will report the baseline characteristics as detailed in Table 2. Linear regression will be employed for analysing continuous outcomes, with transformations applied when necessary. Tobit regression will be done for censored variables. Binomial and logistic regression will be used to analyse binary outcomes. Other types of analysis including generalised regression models or non-parametric analysis may be considered for variables that do not follow a log-normal distribution.

**Table 2:** Characteristics of the study population at baseline (intention-to-treat population)

Baseline variable	Haem iron	Ferrous sulphate
*n*, as randomised	*n*	*n*
Age, months	–	–
Sex (male/female)	–	–
Number of live children born to the mother	–	–
Serum iron, μmol/L	–	–
Serum ferritin, μg/L	–	–
Serum soluble transferrin receptor, mg/L	–	–
Serum sTfR/log_10_(ferritin) index	–	–
Serum transferrin, g/L	–	–
UIBC, μmol/L	–	–
TSAT, %	–	–
Haemoglobin, g/dL	–	–
MCV, fL	–	–
MCH, pg	–	–
MCHC, g/dL	–	–
Reticulocyte count, × 10^9^/L	–	–
Reticulocyte count	–	–
Immature reticulocyte fraction	–	–
Reticulocyte haemoglobin, pg	–	–
Serum hepcidin, μg/L	–	–
Serum erythropoietin, IU/L	–	–
Serum *α*_1_-acid glycoprotein, g/L	–	–
Serum erythroferrone, μg/L	–	–
Below or equal to LOQ (≤ 0.16 μg/L)	–	–
Serum C-reactive protein	–	–
Below or equal to LOQ (≤ 0.60 mg/L)	–	–
Height-for-age *z*-score, SD	–	–
Weight-for-age *z*-score, SD	–	–
Weight-for-height *z*-score, SD	–	–

Values indicate *n*, mean (SD); *n*, geometric mean (geometric SD); *n* (%), prevalence; *n*, median (25 and 75% percentiles)

*LOQ* limit of quantification, *MCH* mean corpuscular haemoglobin, *MCHC* mean corpuscular haemoglobin concentration, *MCV* mean corpuscular volume, *sTfR* soluble transferrin receptor, *TSAT* transferrin saturation, *UIBC* unsaturated iron-binding capacity

The primary analysis concerns the effects of the intervention on haemoglobin concentration and serum ferritin concentration at day 84 after the start of the intervention (day 0), adjusting for the same variable measured at baseline. The analysis will be based on a modified intention-to-treat approach, including all randomised children who received at least one supplemental dose. Effect sizes will be reported with 95% CIs as detailed in Table 3.

**Table 3:** Effect of iron supplementation on primary and secondary outcomes (intention-to-treat population)

Outcome variable	Haem iron	Ferrous sulphate	Group difference, unadjusted (95% CI)	*p*-value	Group difference, adjusted (95% CI)	*p*-value
Serum iron, μmol/L	–	–	–	–	–	–
Serum ferritin, μg/L	–	–	–	–	–	–
Serum soluble transferrin receptor, mg/L	–	–	–	–	–	–
Serum transferrin, g/L	–	–	–	–	–	–
UIBC, μmol/L	–	–	–	–	–	–
TSAT	–	–	–	–	–	–
Serum sTfR/log_10_(ferritin) index	–	–	–	–	–	–
Haemoglobin, g/dL	–	–	–	–	–	–
Anaemia (haemoglobin < 11.0 g/dL)	–	–	–	–	–	–
MCV, fL	–	–	–	–	–	–
MCH, pg	–	–	–	–	–	–
MCHC, g/dL	–	–	–	–	–	–
Reticulocyte count, × 10^9^/L	–	–	–	–	–	–
Reticulocyte count, %	–	–	–	–	–	–
Immature reticulocyte fraction, %	–	–	–	–	–	–
Reticulocyte haemoglobin, pg	–	–	–	–	–	–
Serum hepcidin, μg/L	–	–	–	–	–	–
Serum erythropoietin, IU/L	–	–	–	–	–	–
Serum erythroferrone, μg/L	–	–	–	–	–	–
Serum C-reactive protein, mg/L	–	–	–	–	–	–
Serum *α*_1_-acid glycoprotein, g/L	–	–	–	–	–	–
Height-for-age *z*-score, SD	–	–	–	–	–	–
Weight-for-age *z*-score, SD	–	–	–	–	–	–
Height-for-weight *z*-score, SD	–	–	–	–	–	–

In secondary analyses, we will adjust intervention effects for sex, age, haemoglobin concentration and serum concentrations of ferritin and soluble transferrin receptor at baseline (continuous variables except sex). Fractional polynomial regression analysis will be utilised to explore the relationship between the magnitude of the intervention effect on haemoglobin levels and the baseline iron status.

#### Methods in analysis to handle protocol non-adherence and any statistical methods to handle missing data {20c}

In the primary analysis, multiple imputation will be used if more than 5% of the data are missing. Imputation will be done separately within each intervention group, utilising predictive mean matching based on the nearest three neighbouring values. The imputation model will incorporate all variables from the dataset except censored variables and variables derived from original data, such as body mass index (calculated from height and length) and transferrin saturation (to be derived from serum concentrations of iron and UIBC). We will employ a process involving at least 20 imputations, to be conducted iteratively through chained equations. Additionally, a per-protocol analysis will be performed to determine the sensitivity of the intention-to-treat analysis. The per-protocol analysis will be a comparison of the treatment groups that include only those patients who completed the treatment originally allocated and have endpoint measurement.

#### Methods for additional analyses (e.g. subgroup analyses) {20b}

We will use multiple fractional polynomial regression analysis to explore to what extent group differences in haemoglobin concentration depend on iron status at baseline (as indicated by serum ferritin concentration) and on inflammation at baseline (as indicated by serum concentrations of CRP and AGP). When there is statistical support for such effect size modification, we will report intervention effects with 95% CIs.

Adverse events will be analysed per protocol analysis. The number of adverse events and type of adverse event by treatment group will be reported as detailed in Table 4. If the number of reported adverse events is less than 20 per adverse event type per intervention group, no formal statistical procedure will be conducted. For adverse effects that occur relatively frequently (more than 20 per intervention arm), we will evaluate the group differences in the incidence of adverse events using Poisson or negative binomial regression models with zero inflation as appropriate.

**Table 4:** Number of adverse events by treatment type (per-protocol analysis)

Diagnosis	Haem iron	Ferrous Sulphate
**Respiratory**	–	–
Upper respiratory tract infection	–	–
Pneumonia	–	–
Bronchiolitis	–	–
**Gastrointestinal**	–	–
Gastroenteritis	–	–
Acute watery diarrhoea	–	–
Persistent diarrhoea	–	–
Bacillary dysentery	–	–
**Skin**	–	–
Skin infection	–	–
Abscess	–	–
Conjunctivitis	–	–
Total number of AE	–	–

### Interim analyses {21b}

There is no interim analysis planned for this study.

### Dissemination plans {31a}

This study will be exclusively conducted in rural Gambia, where the Medical Research Council Unit The Gambia (MRCG) has been actively involved for several years. Building upon the successful completion of another iron supplement study in the same area [16], which enjoyed high participation rates, we have established a strong relationship with the local population. Recognising the significant contributions made by the Gambian people to medical science in general and to our nutritional studies and considering the direct relevance of this study’s findings to them, we have made a commitment to prioritise our public engagement efforts locally.

When undertaking any new research, we engage with the villagers through sensitisation meetings that precede individual-level information and consenting procedures. These meetings serve as an opportunity to share summaries of outcomes from previous and ongoing studies before introducing the plan for the new study. This continuous flow of information allows us to maintain an ongoing dialogue with the community. Additionally, we regularly organise open days to foster interaction and knowledge sharing.

Furthermore, we collaborate closely with the National Nutrition Agency (NaNA) in Gambia. We have established close ties with other relevant government departments, as well as with the local offices of the World Health Organization (WHO) and UNICEF.

Any request for the use of study data will go through approval from the sponsor and the ethics committee. All data will be in an anonymous format for external users. Data sharing will agree with the sponsor policy on research data sharing {31c}.

## Discussion

This study will determine if supplementation with haem iron is more efficacious than with conventional ferrous sulphate in enhancing haemoglobin and ferritin concentrations in anaemic children aged 6–12 months. It will potentially provide support for the hypothesis that haem iron is absorbed through molecular pathways that are not affected by inflammation. Slaughterhouses generate substantial volumes of blood as by-products during meat production, presenting an opportunity for local production of accessible and cost-effective haem iron products. Therefore, if proven efficacious and safe, the implications of this research could extend beyond individual health benefits potentially leading to policy changes.

Although daily supplementation for 84 days presents a potential adherence challenge, we can draw confidence from the high adherence rates reported in our previous trial conducted in the same study location [16]. To further enhance adherence, we will implement the following strategies:

- Participant selection: We will enrol participants who do not have plans to travel during the study period and are committed to staying within their communities. This will help minimise disruptions and ensure their availability for the entire duration of the trial.
- Daily field visits: Our dedicated field staff will visit participants daily to administer the supplements and closely monitor their progress. This regular interaction will not only reinforce adherence but also provide an opportunity to address any concerns or challenges participants may encounter.
- Ongoing monitoring and support: We will establish a robust system for monitoring daily field activities and conducting biweekly meetings with the field staff. This proactive approach will allow us to identify and address any adherence issues promptly, ensuring that the study protocol is followed consistently.
- Incentives for participation: We recognise the commitment required from participants and the potential impact on their daily routines. To compensate for their loss of earnings during baseline and endpoint assessments, we will provide appropriate compensation. Additionally, participants who need to travel during the study will be called for endpoint assessments, and their travel expenses will be reimbursed as a motivation for their continued involvement.

## Trial status

The protocol for this study, denoted as version 1.2 (dated August 2022) and version 2.0 (dated 16 May 2023), received approval from the Gambia Government/MRC Joint Ethics Committee {11}. The enrolment of participants began on 11 May 2023, and we anticipate completing the final participant’s last visit by 31 March 2024.

### Supplementary Information

**Supplementary Material 1.**

**Supplementary Material 2.**

**Supplementary Material 3.**

**Supplementary Material 4.**

**Supplementary Material 5.**

## References

[1] Abdelazim IA, Abu-Faza M, Shikanova S, Zhurabekova G, Maghrabi MM (2018). Heme-bound iron in treatment of pregnancy-associated iron deficiency anemia. J Family Med Prim Care [Internet]..

[2] Suchdev PS, Jefferds MED, Ota E, da Silva LK, De-Regil LM. Home fortification of foods with multiple micronutrient powders for health and nutrition in children under two years of age. Cochrane Database Syst Rev [Internet]. 2020;2020(2) [cited 2023 Oct 11]; Available from: https://www-cochranelibrary-com.ezproxy.library.wur.nl/cdsr/doi/10.1002/14651858.CD008959.pub3/full. 10.1002/14651858.CD008959.pub3 PMC7046492 32107773

[3] Prentice AM, Bah A, Jallow MW, Jallow AT, Sanyang S, Sise EA, et al. Respiratory infections drive hepcidin-mediated blockade of iron absorption leading to iron deficiency anemia in African children. Sci Adv [Internet]. 2019;5(3) [cited 2023 Jun 7] Available from: /pmc/articles/PMC6436921/. 10.1126/sciadv.aav9020 PMC6436921 30944864

[4] Wegmüller R, Bah A, Kendall L, Goheen MM, Mulwa S, Cerami C (2016). Efficacy and safety of hepcidin-based screen-and-treat approaches using two different doses versus a standard universal approach of iron supplementation in young children in rural Gambia: a double-blind randomised controlled trial. BMC Pediatr [Internet]..

[5] Bah A, Wegmuller R, Cerami C, Kendall L, Pasricha SR, Moore SE, et al. A double blind randomised controlled trial comparing standard dose of iron supplementation for pregnant women with two screen-and-treat approaches using hepcidin as a biomarker for ready and safe to receive iron. BMC Pregnancy Childbirth [Internet]. 2016;16(1) [cited 2023 Jun 6]. Available from: /pmc/articles/PMC4944263/. 10.1186/s12884-016-0934-8 PMC4944263 27411564

[6] Bjorn Rasmussen E, Hallberg L, Isaksson B, Arvidsson B (1974). Food iron absorption in man. Applications of the two-pool extrinsic tag method to measure heme and nonheme iron absorption from the whole diet. J Clin Investig [Internet]..

[7] Wheal MS, Decourcy-Ireland E, Bogard JR, Thilsted SH, Stangoulis JCR (2016). Measurement of haem and total iron in fish, shrimp and prawn using ICP-MS: implications for dietary iron intake calculations. Food Chem..

[8] Young MF, Griffin I, Pressman E, McIntyre AW, Cooper E, McNanley T (2010). Utilization of iron from an animal-based iron source is greater than that of ferrous sulfate in pregnant and nonpregnant women. J Nutr [Internet]..

[9] Zenke-Kawasaki Y, Dohi Y, Katoh Y, Ikura T, Ikura M, Asahara T (2007). Heme induces ubiquitination and degradation of the transcription factor Bach1. Mol Cell Biol [Internet]..

[10] Zhou Y, Wu H, Zhao M, Chang C, Lu Q (2016). The Bach family of transcription factors: a comprehensive review. Clin Rev Allergy Immunol..

[11] WHO (2023). Diarrhoea [Internet].

[12] WHO (2011). Haemoglobin concentrations for the diagnosis of anaemia and assessment of severity [Internet].

[13] WHO. WHO guideline on use of ferritin concentrations to assess iron status in individuals and populations [Internet]. Geneva; 2020. [cited 2023 Sep 9]. Available from: https://apps.who.int/iris/bitstream/handle/10665/331505/9789240000124-eng.pdf. 33909381

[14] Wegmüller R, Bah A, Kendall L, Goheen MM, Sanyang S, Danso E (2023). Hepcidin-guided screen-and-treat interventions for young children with iron-deficiency anaemia in The Gambia: an individually randomised, three-arm, double-blind, controlled, proof-of-concept, non-inferiority trial. Lancet Glob Health [Internet]..

[15] Wegmüller R, Bah A, Kendall L, Goheen MM, Sanyang S, Danso E (2023). Hepcidin-guided screen-and-treat interventions for young children with iron-deficiency anaemia in The Gambia: an individually randomised, three-arm, double-blind, controlled, proof-of-concept, non-inferiority trial. Lancet Glob Health [Internet]..

[16] Bah M, Stelle I, Verhoef H, Saidykhan A, Moore SE, Susso B, Prentice AM, Cerami C (2024). Early iron supplementation in exclusively breastfed Gambian infants: a randomized controlled trial. Bull World Health Organ..

