# Supplementary material for: Haem iron versus ferrous iron salts to treat iron deficiency anaemia in Gambian children: protocol for randomised controlled trial {1}
Source: Trials. 2024 Apr 19;25:270. doi: 10.1186/s13063-024-08101-0 (PMC11027386; doi:10.1186/s13063-024-08101-0)
Supplement: Supplementary file 1 — Supplementary Material 1. [file 13063_2024_8101_MOESM1_ESM.docx]

**Additional File 1:**

**Supplementary Table 1:** Villages in Jarra West and Kiang East which were included in the recruitment area.

| **Village Names** | |
| --- | --- |
| **Jarra West** | **Kiang East** |
| Misera | Massembeh |
| Sare Musa | Geniere |
| Sey Kunda | Kolior |
| Jiffin | Madina kaba Kunda |
| Tonyataba | Kiang Sare Musa |
| Sankwia | Jomari |
| Pakalinding | Sare Pateh |
| Karantaba | Kaiaf |
| Jabisa | Yoro Jula |
| Soma | Jasobo |
| Sumbundou |  |
| Sare Biran |  |
| Medina Fonkoi |  |
| Kohel |  |
| Sare Saidy |  |
| Genoi |  |
| Seno Bajonki |  |
| Fonkoi Kunda |  |
| Kani kunda |  |
| Si Kunda |  |
