# Supplementary material for: Haem iron versus ferrous iron salts to treat iron deficiency anaemia in Gambian children: protocol for randomised controlled trial {1}
Source: Trials. 2024 Apr 19;25:270. doi: 10.1186/s13063-024-08101-0 (PMC11027386; doi:10.1186/s13063-024-08101-0)
Supplement: Supplementary file 2 — Supplementary Material 2. [file 13063_2024_8101_MOESM2_ESM.docx]

**Additional file 2:**

**PARTICIPANT INFORMATION SHEET**

| Version | 2.1 | Date | 14 August 2023 |
| --- | --- | --- | --- |

Study Title: Haem iron to combat iron deficiency in anaemic Gambian children: A Double Blind Randomised Controlled Trial **(IDeA study 3)**

| SCC/Protocol No: | 27648 |
| --- | --- |

Sponsor & Funder:

What is informed consent?

You are invited to let your child take part in a research study. Before you decide, you need to understand why the study is being done and what will happen in it. Please take time to read the following information or get the information explained to you in your language. Listen carefully. You can ask questions if there is anything that is not clear, or you do not understand. You may also wish to speak to your husband/wife, family members, friends, or others before deciding to let your child take part in the study.

If you decide to allow your child to join the study, you will need to sign or put a thumbprint on a consent form saying you agree for your child to be in the study. You will receive a copy of the consent form.

Why is this study being done?

As you know many children have “anaemia” (sickness that makes someone have less blood), and the number of children with this sickness is very high in Gambia. We know that giving children a medicine called “iron” treats and prevents someone from “anaemia”. There is strong evidence to suggest that “anaemia” can impair the physical and developmental potential of children, especially in early childhood. This trial is being done to find out if iron in the form of haem iron is more effective than the usual source of iron that is used. Haem iron is the kind that is found in meat. We are testing a haem iron product that is derived from cows and is commonly used in the United States. We hope to discover that children in The Gambia will recover from their anaemia if they are given haem iron drops.

Please ask us any questions that you do not understand. If you would like more information, we are happy to explain this to you more than once.

We will tell the results of this study to your community.

What is the new vaccine/drug?

“Iron” in the form of Haem iron. This is an iron supplement commonly used and sold in The United States.

What does this study involve?

The following will be required from your child if you agree to take part:

Your child’s health will be examined and his/her height and weight will be measured. If your child is enrolled in the study, he/she will be randomly allocated to one of the two study groups:

Group A: Ferrous sulphate iron (the type we usually give in the clinic).

Group B: Haem iron (like the type found in meat).

Your child will then take one of the supplements every day for 12 weeks.

We will draw a small amount of blood (3.5ml) from your child at enrolment and then again after 84 days of supplementation.

We will also collect a stool sample from your child at enrolment and then at the end of the supplement period.

One of our study staff will visit you daily and to give your child the iron supplements. Once per week, our study staff will ask you questions about what liquids and food your child is drinking and eating.

After the end of the 12 weeks of supplementation, you will still be provided with medical care by our clinical team for two weeks. All you have to do is to contact the study team once your child is unwell. The clinical team will either visit or invite you to come to the clinic for further assessment and will decide on treatment/referral option.

If we find out that your child is sick and decide that he/she cannot join the study because of that, he/she will receive the care routinely available in The Gambia. Your child may be treated at the study site and if necessary, referred to a health facility that can manage the condition better.

If the research study needs to be stopped, we will tell you and your child will have the normal medical care if needed.

What will happen to the samples taken in this study?

The collected blood and stool samples will be transported to the MRCG laboratories. There we will test the levels of iron and of essential components in your child’s blood and stool that are relevant for the study. In addition, we will assess markers in the blood that may indicate that your child has an infection. We will also store the samples for further analysis of other markers that may indicate side effects of iron supplements. Some of the blood or stool samples, including the DNA, may be transferred to laboratories overseas for analysis because we don’t have the equipment required for measuring all the factors we are investigating in The Gambia.

What harm or discomfort can you expect in the study?

Collection of blood can cause discomfort, but it will not cause any harm to your child. There is a chance that your child may get a bruise from where we took the blood.

There is a small risk that iron supplements can cause diarrhoea or other tummy discomfort. Also, there is evidence that exposure to meat product could lead to allergic reactions to some individuals. There is limited evidence with regards to the prevalence of this reaction in our setting. We anticipate minimal adverse events associated with meat allergies. However, this will be closely monitored by our clinical team.

What benefits can you expect in the study?

By participating in this study your child will receive medical care at a level greater than that usually given within the study area. We will closely monitor your child’s health and give you regular feedback.

Will you be reimbursed for your loss of earnings during your child’s/ward’s participation in the study?

You will be given D250 for loss of earnings and time on a baseline and endline day. Also, MRC will provide you breakfast and where required lunch. You will also be provided transport or given back the money for your transport on a baseline and endline day.

Are there other products or treatment?

No.

What happens if you refuse to participate in the study or change your mind later?

You are free to let your child join or not in the study and you are free to stop taking part at any time without giving a reason. You and your child will still get the normal medical care.

If you do not want your child to continue in the study, we will use only the samples and information already collected from your child.

The study doctor may ask to do some tests if needed for your child’s safety.

If we find any new information during the study that may change if your child can be in the study, we will inform you as soon as possible.

What compensation will be available if your child is injured during the study?

We will provide medical care if your child gets any problems from the study through the MRC indemnity arrangements or insurance.

If it is an emergency, please go to your nearest health centre or clinic and call immediately the field worker who gave his/her telephone number to you or contact Dr Emmanuel Okoh on +220 2777870

How your child’s information will be kept and who will be allowed to see it?

All information that is collected about your child in the study will be kept strictly confidential. Your child’s personal information will only be seen by the study team members, the sponsor and if necessary, the Ethics Committee and Government authorities.

We will need to use information from you and your child for this research project. All information collected about you will be kept private. Only the study staff and authorities who check that the study is being carried out properly will be allowed to look at information about you. Information will include your child’s initials/study ID/ number/name/contact details/other identifiers used for the research project. We will keep all information about you safe and secure.

Data may be sent to other study staff in MRC Unit The Gambia at LSHTM, but this will be anonymised. This means that any information about you which leaves the clinic will have your name and address removed so that you cannot be recognised and your data will have a code number instead.

Some of your information will be sent to the United Kingdom. They must follow our rules about keeping your information safe.

Your personal details, meaning your name and other identifiable information, will be kept in a different safe place to the other study information and will be destroyed within 10 years of the end of the study.

At the end of the project, the study data will be archived at MRC Unit The Gambia at LSHTM. The data will be made available to other researchers worldwide for research and to improve medical knowledge and patient care. Your personal information will not be included and there is no way that you can be identified.

You can find out more about how we use your information

- At https://www.lshtm.ac.uk/files/research-participant-privacy-notice.pdf
- by asking one of the research team
- by sending an email to DPO@lshtm.ac.uk

Who should you contact if you have questions?

If you have any questions or are worried you can call Dr Emmanuel Okoh on +220 2777870 or Dr Carla Cerami on +220 787 5756 and you can always also call the personal numbers of the study staff given to you. If you have any concerns, you can also contact staff at your health centre or hospital.

Please feel free to ask any question you might have about the study.

## Who has reviewed this study?

The study has been checked by scientists at the Medical Research Council and by the Gambia Government/MRC Joint Ethics Committee. The Ethics Committee protects your rights and wellbeing and has given permission for it to take place. Additionally, it has been reviewed by the Ethics Committees at The London School of Hygiene and Tropical Medicine and at King’s College London.

**Consent**

Participant’s Name

Participant’s Identification Number: |__|__|__|__|__|__|__|__|__|__|

**OR**

(Printed name of parent) (Printed name of guardian)

I have read the written information **OR**

I have had the information explained to me by study personnel in a language that I understand

and I

- confirm that my choice to let my child participate is entirely voluntarily,
- confirm that I have had the opportunity to ask questions about this study and I am happy with the answers that have been provided,
- understand that I allow access to the information about my child by the persons described in the information sheet,
- had enough time to think about whether I want my child take part in this study
- agree to allow my child take part in this study.

*Tick as appropriate*

| I agree for my child’s samples to be shipped outside the Gambia.  I agree to further research on my child’s samples including genetic testing | | | | Yes  Yes | No  No | |
| --- | --- | --- | --- | --- | --- | --- |
|  |  |  |  | | | |
| Participant’s parent/guardian signature/thumbprint* |  |  |  | | |  |
|  |  |  | Date Time | | | |
| Printed name of impartial witness* |  | | | | | |
| Signature of impartial witness* |  |  |  | | |  |
|  |  |  | Date Time | | | |
| Printed Name of Person obtaining consent |  | | | | | |
| **I attest that I have explained the study information accurately in** _______________________ **and was understood to the best of my knowledge by, the participant/parent/guardian and that he/she has freely given consent to participate ***in the presence of the above named impartial witness (where applicable).** A copy of this ICF has been provided to the participant. | | | | | | |
| Signature of Person obtaining consent |  |  |  | | | |
|  |  |  | Date (dd/mmm/yyyy) Time (24hr) | | | |

**Only required if the participant is unable to read or write.*
