# Supplementary figures and images for: Haem iron versus ferrous iron salts to treat iron deficiency anaemia in Gambian children: protocol for randomised controlled trial {1}
Source: Trials. 2024 Apr 19;25:270. doi: 10.1186/s13063-024-08101-0 (PMC11027386; doi:10.1186/s13063-024-08101-0)

**Additional File 3: PACTR Registration Listing**


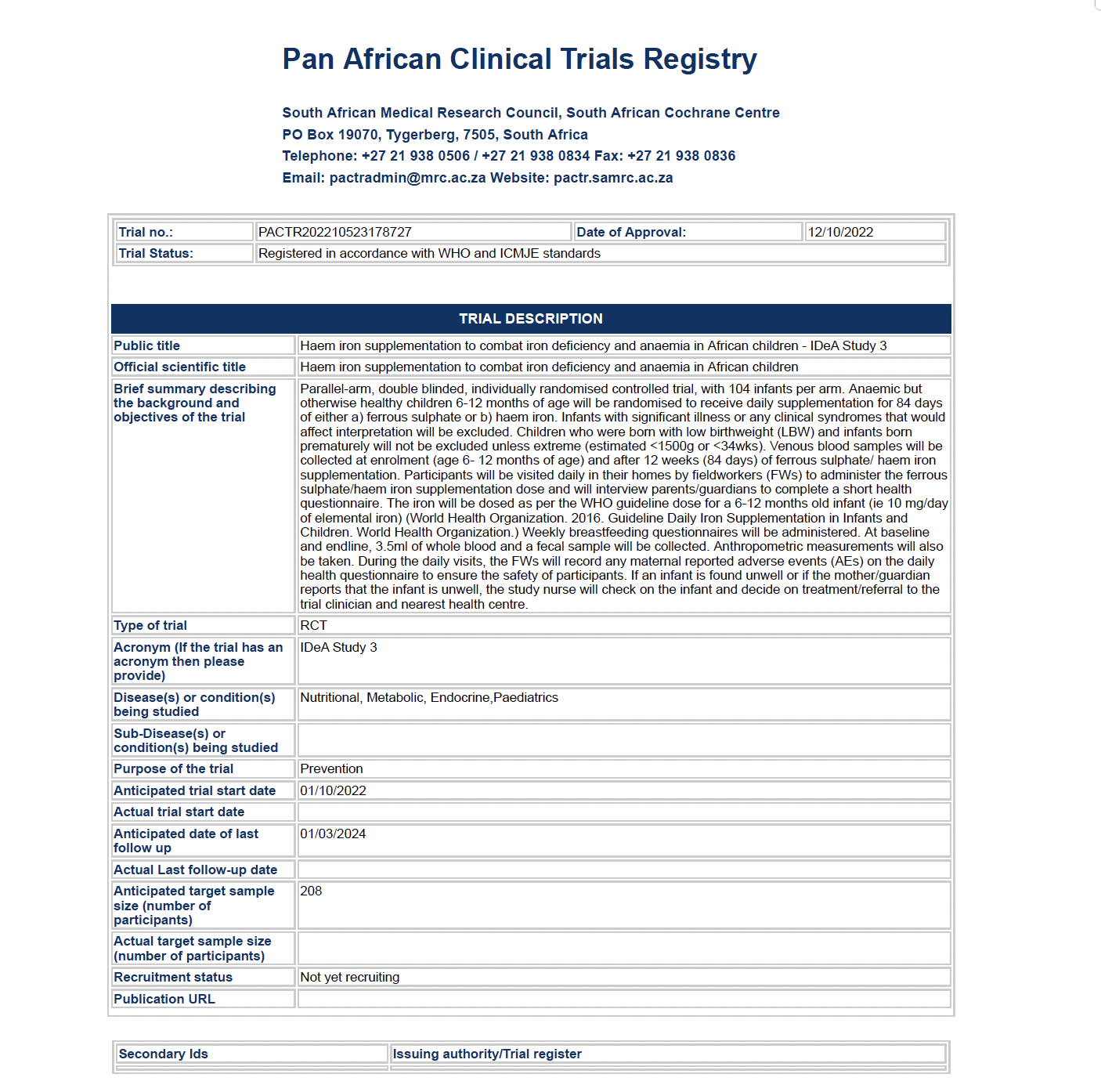


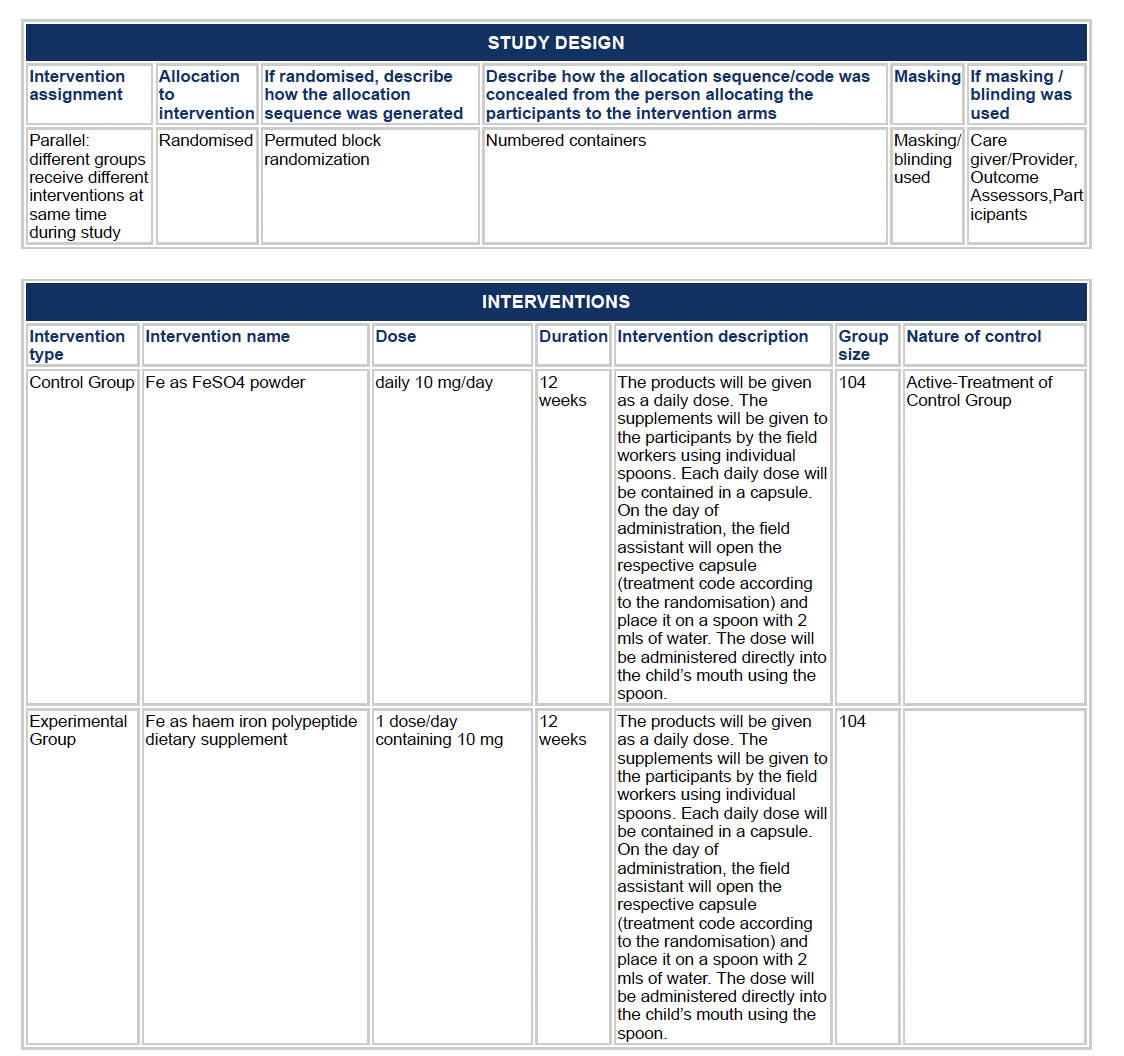


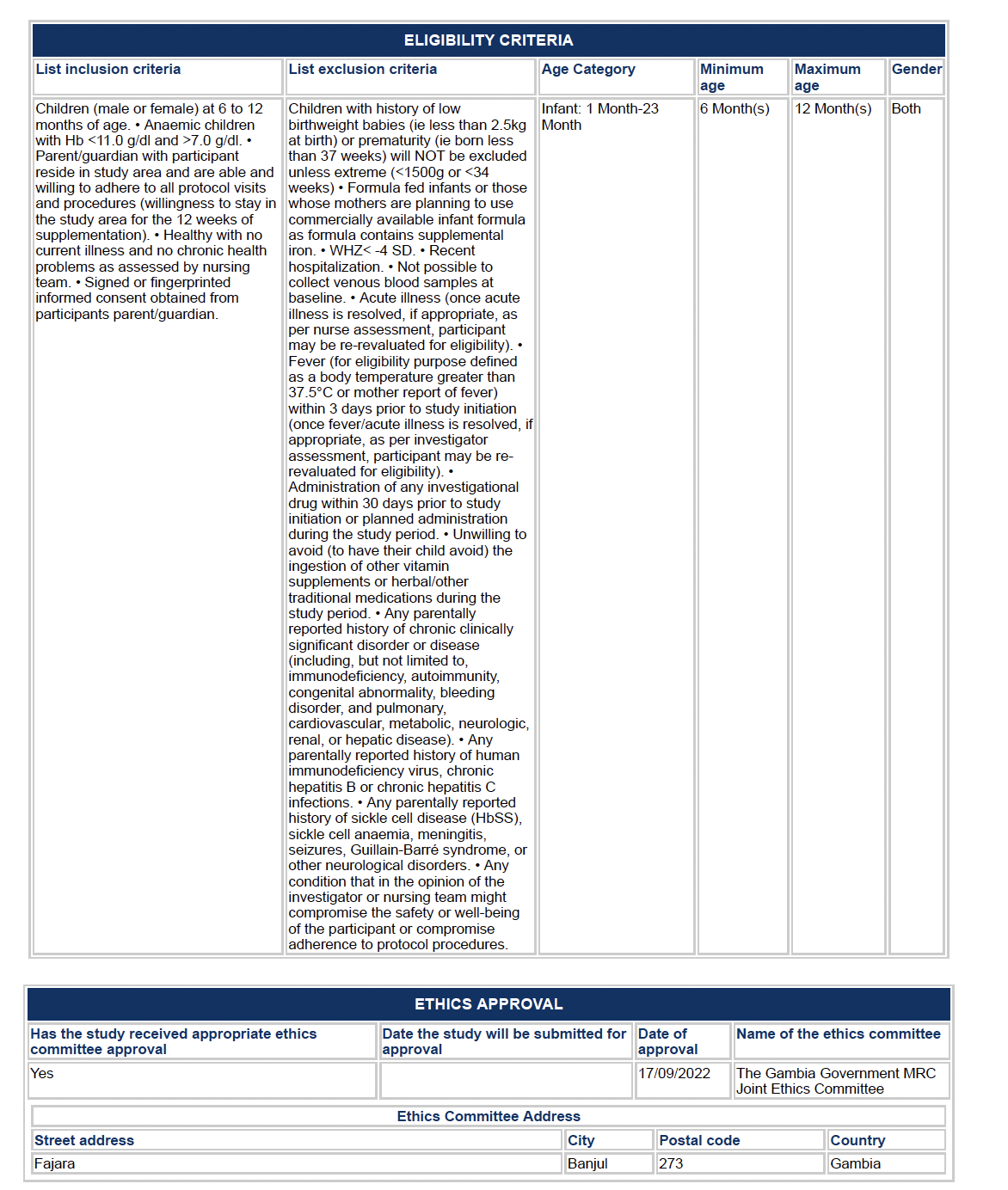


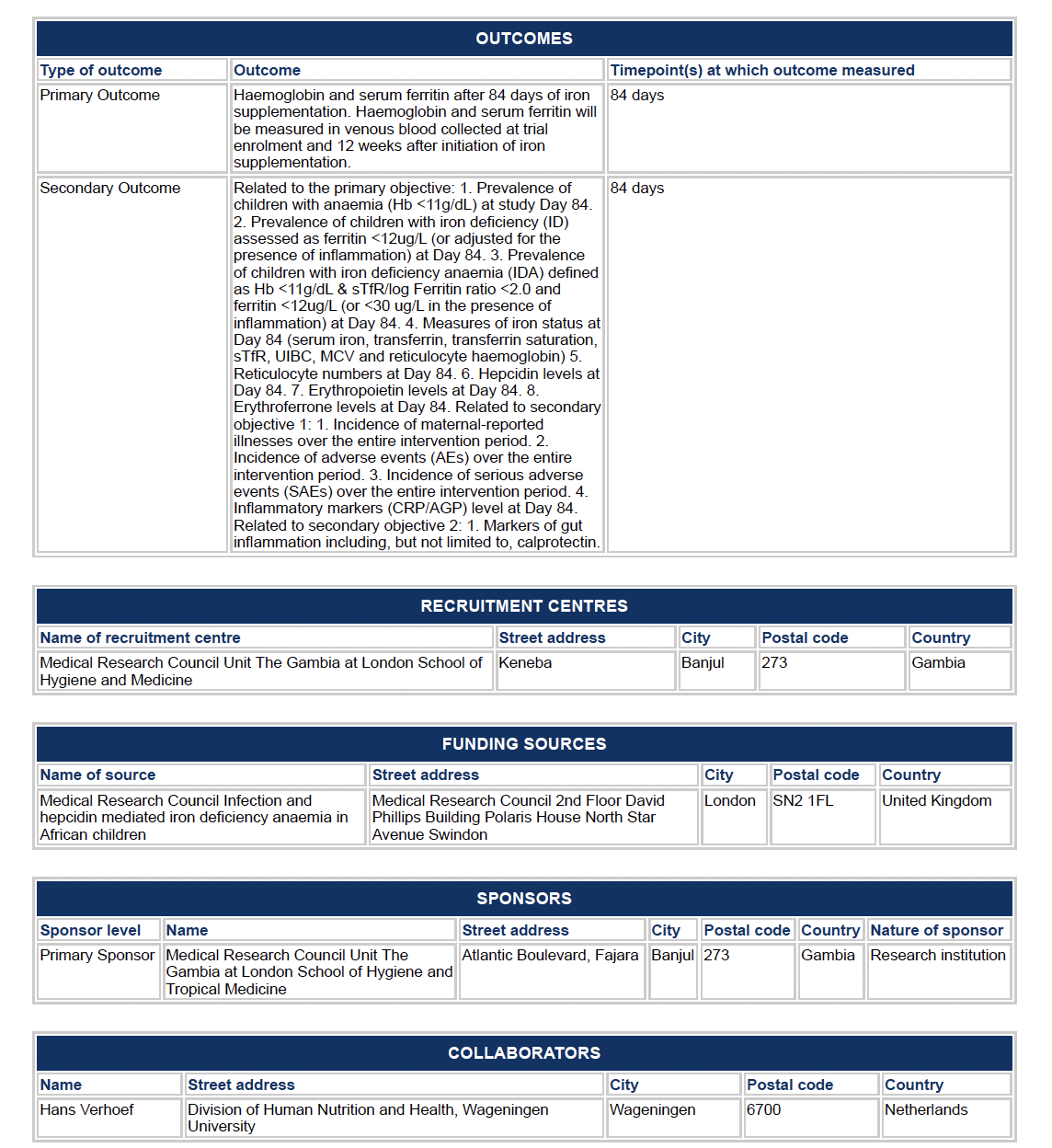


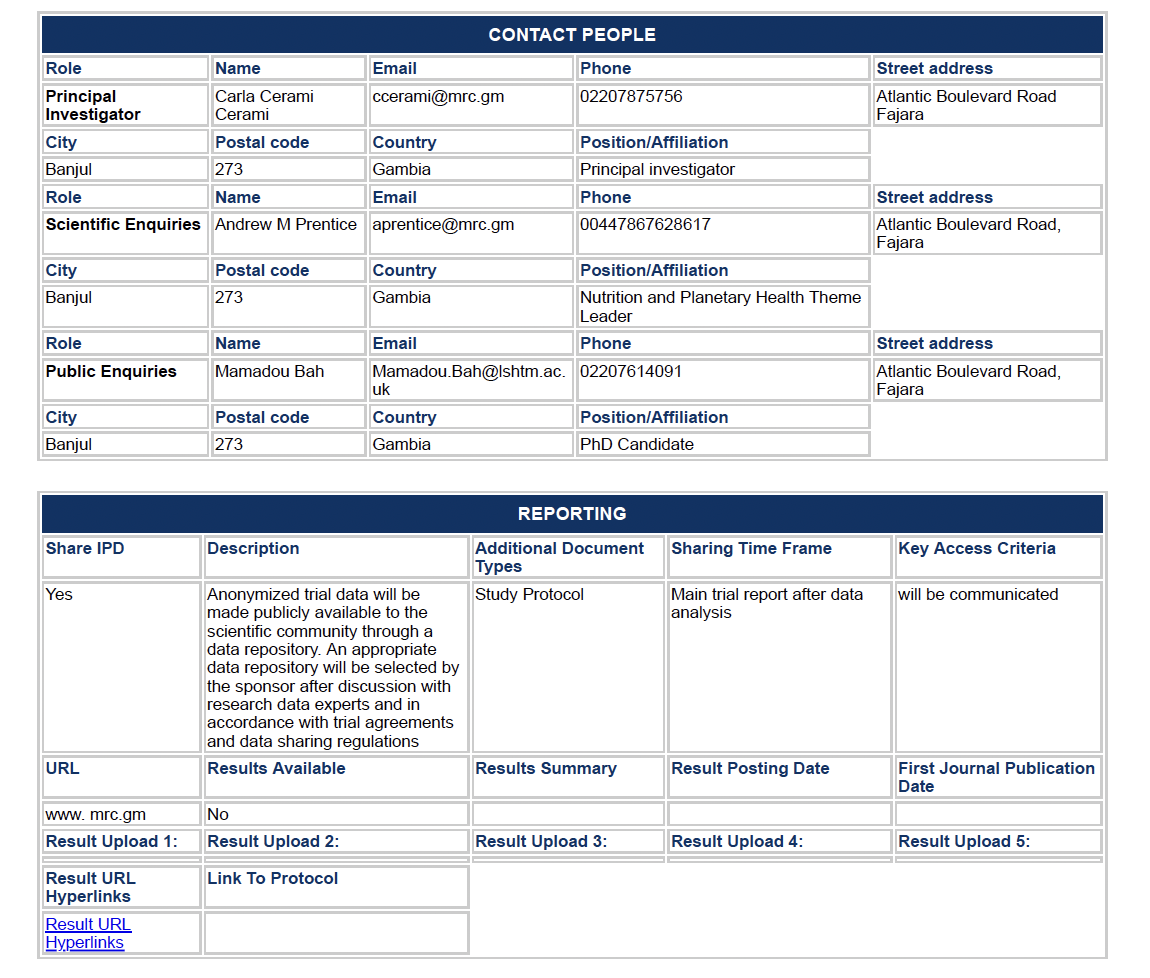

Supplement: Supplementary file 3 — Supplementary Material 3. [file 13063_2024_8101_MOESM3_ESM.docx]

**Additional File 4: Adverse Events eCRF**
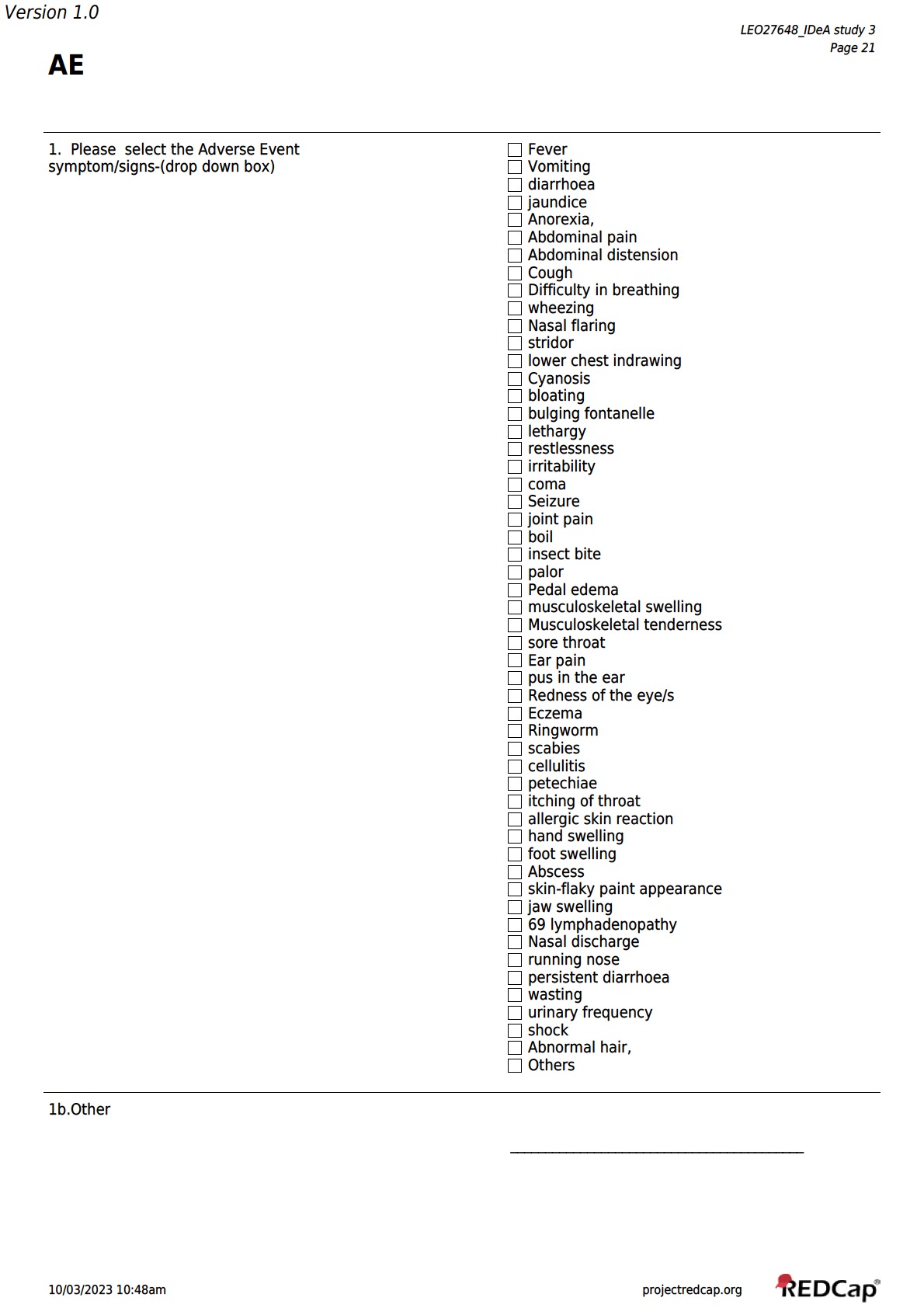


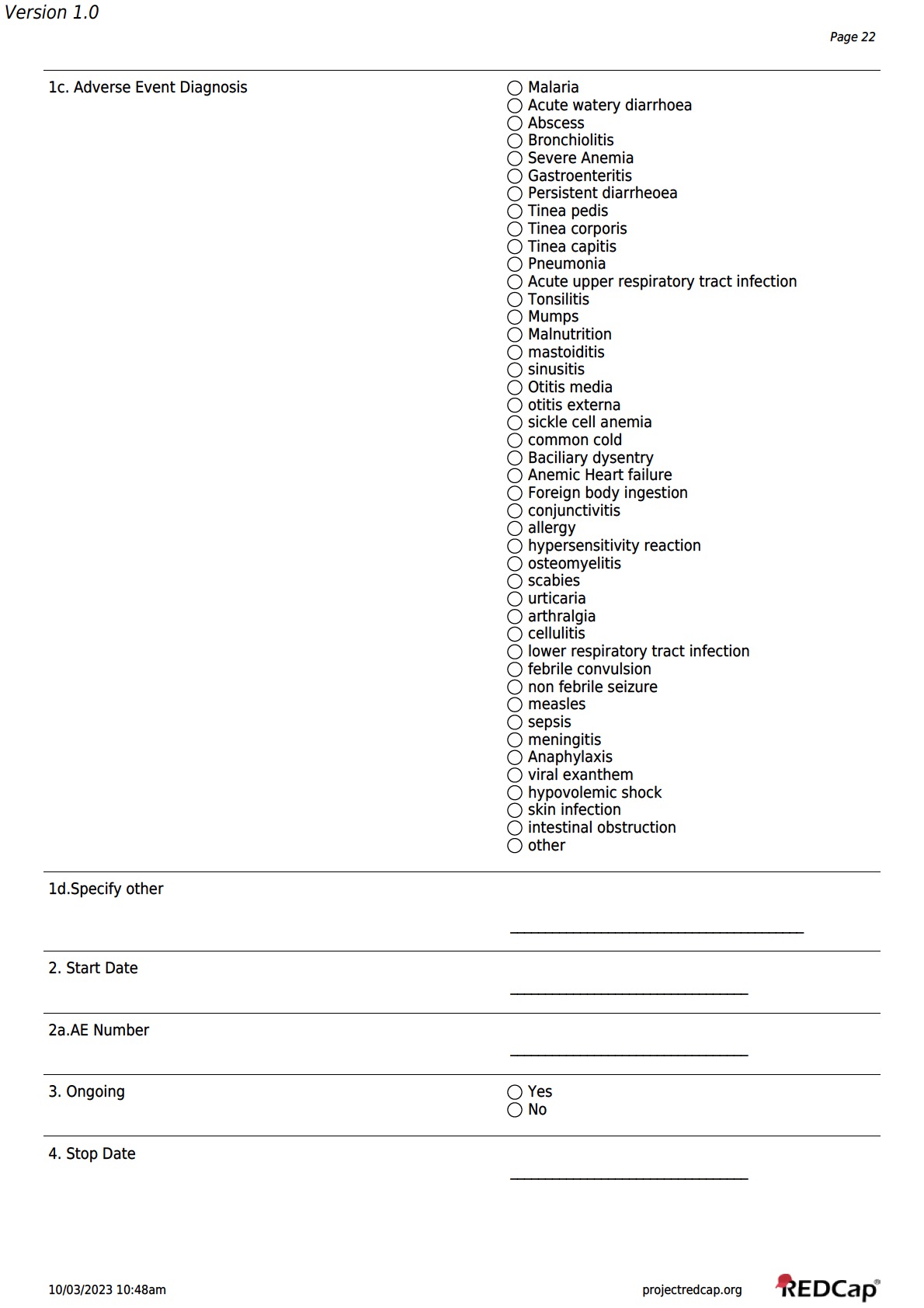

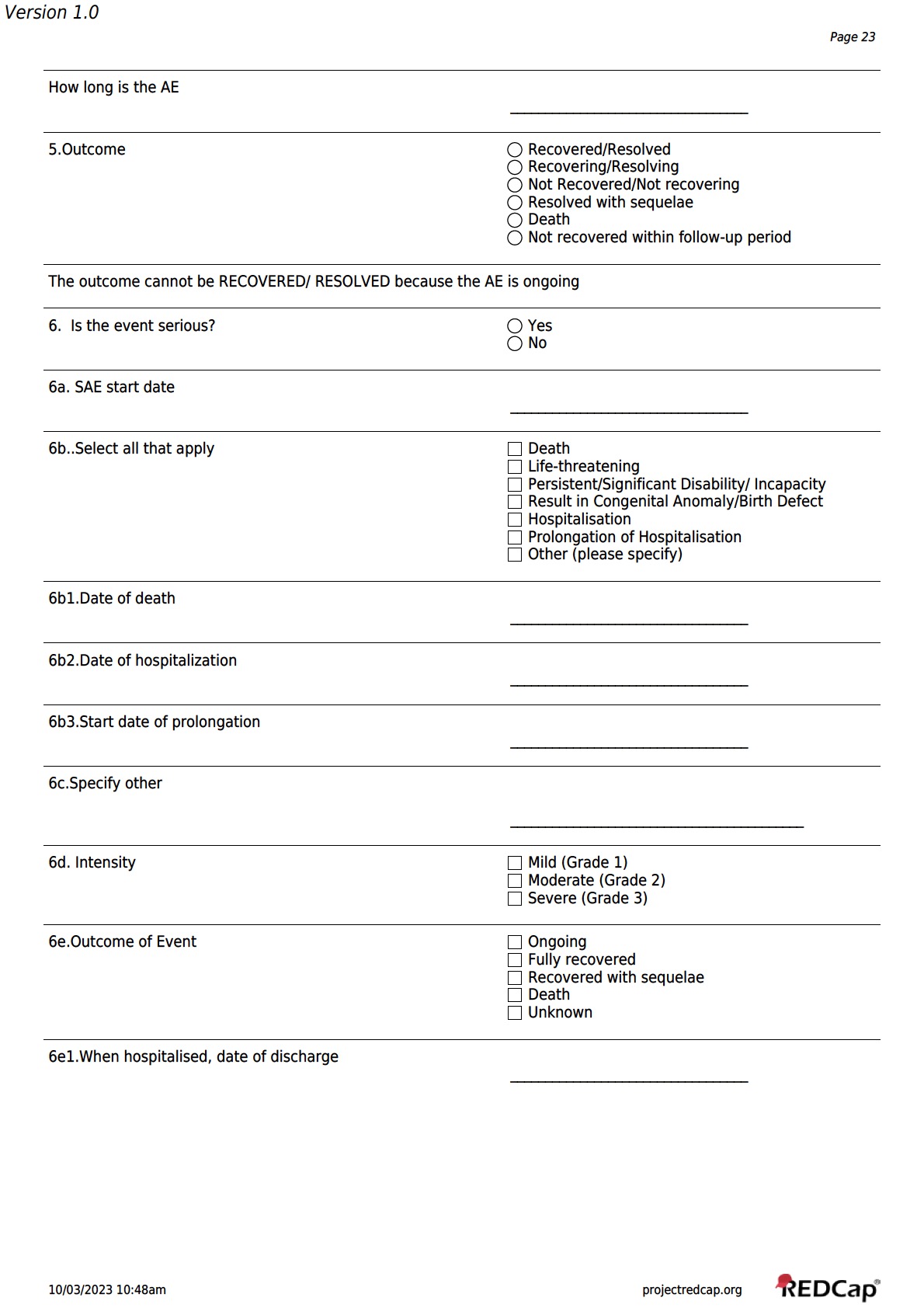

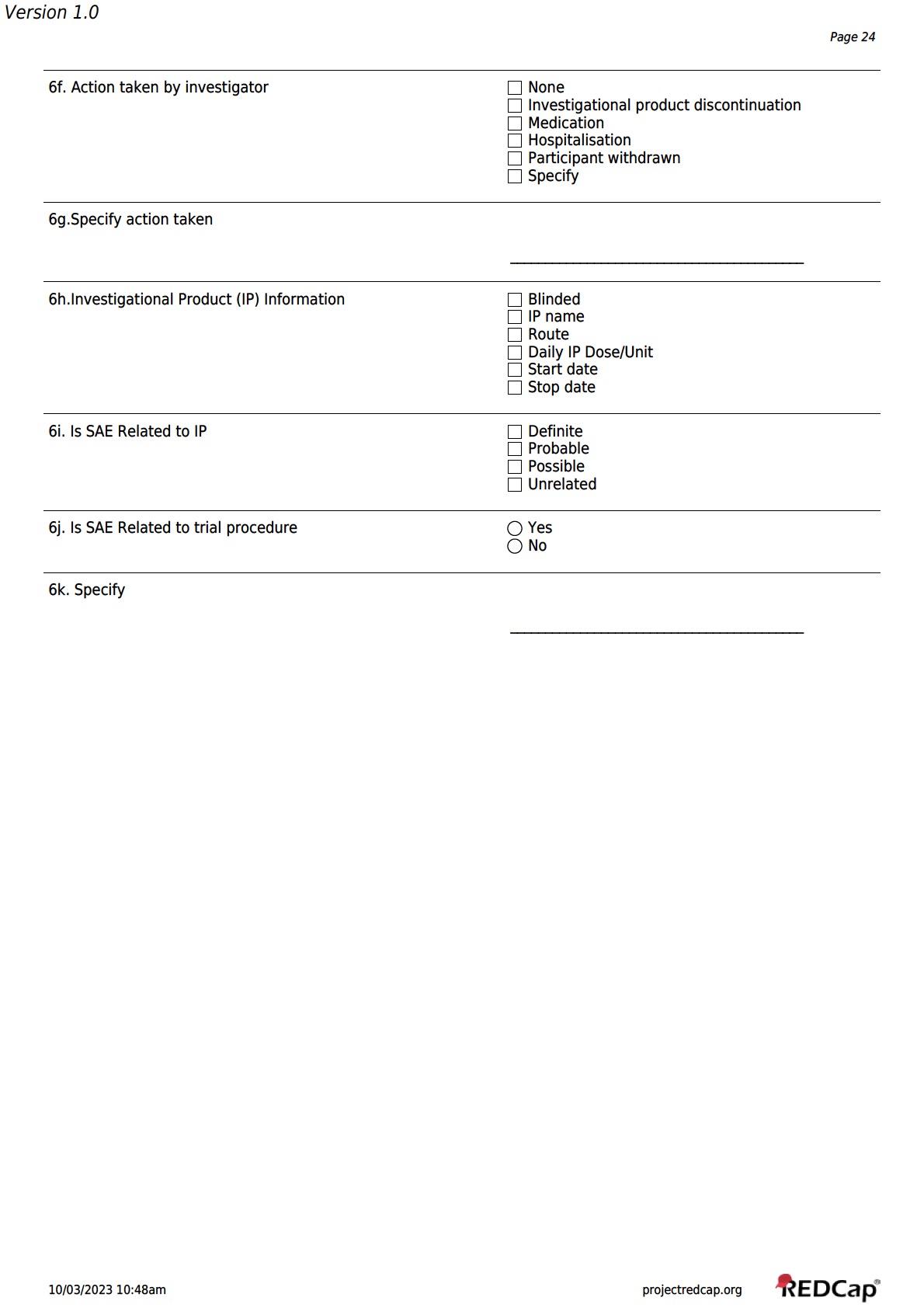

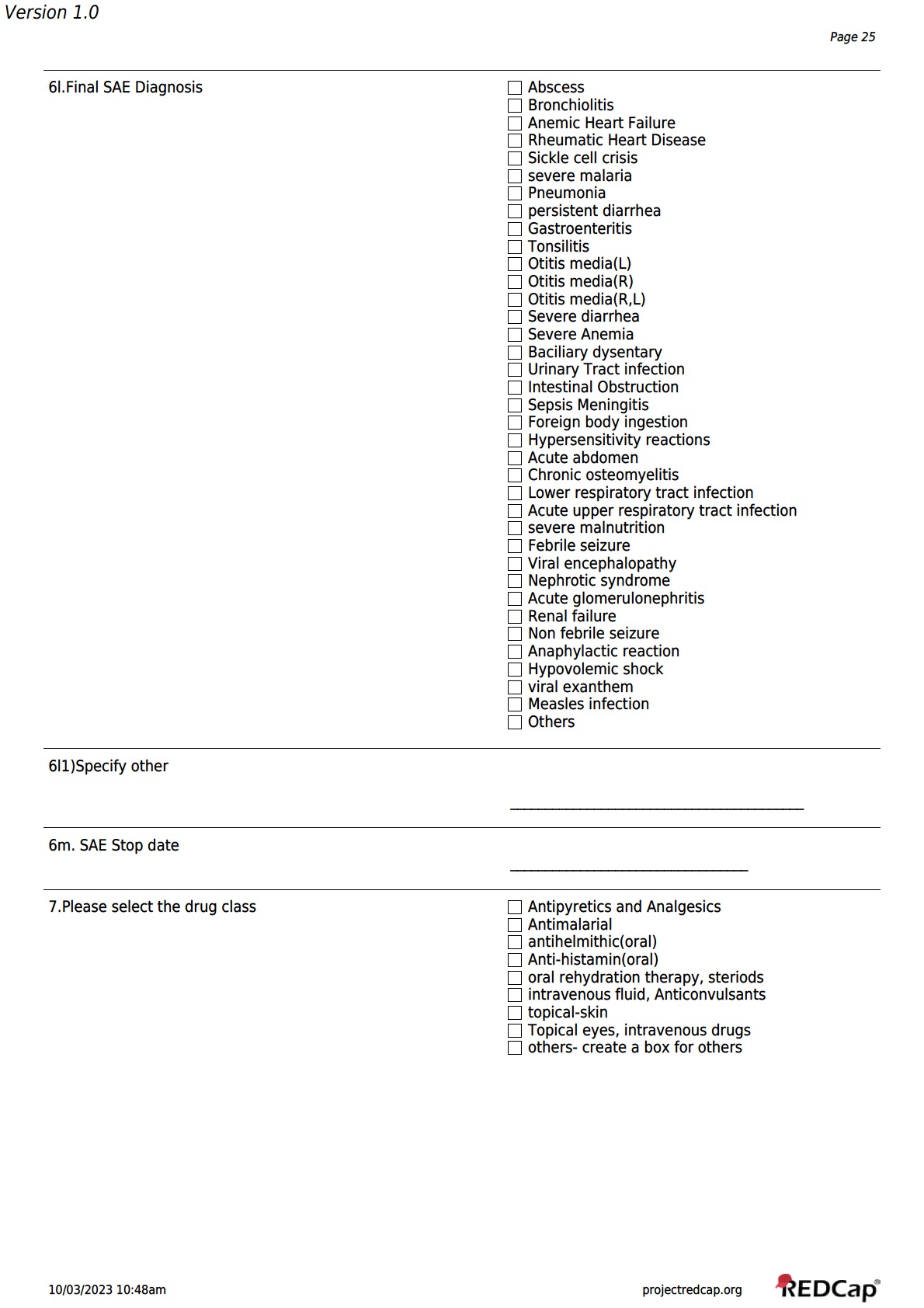

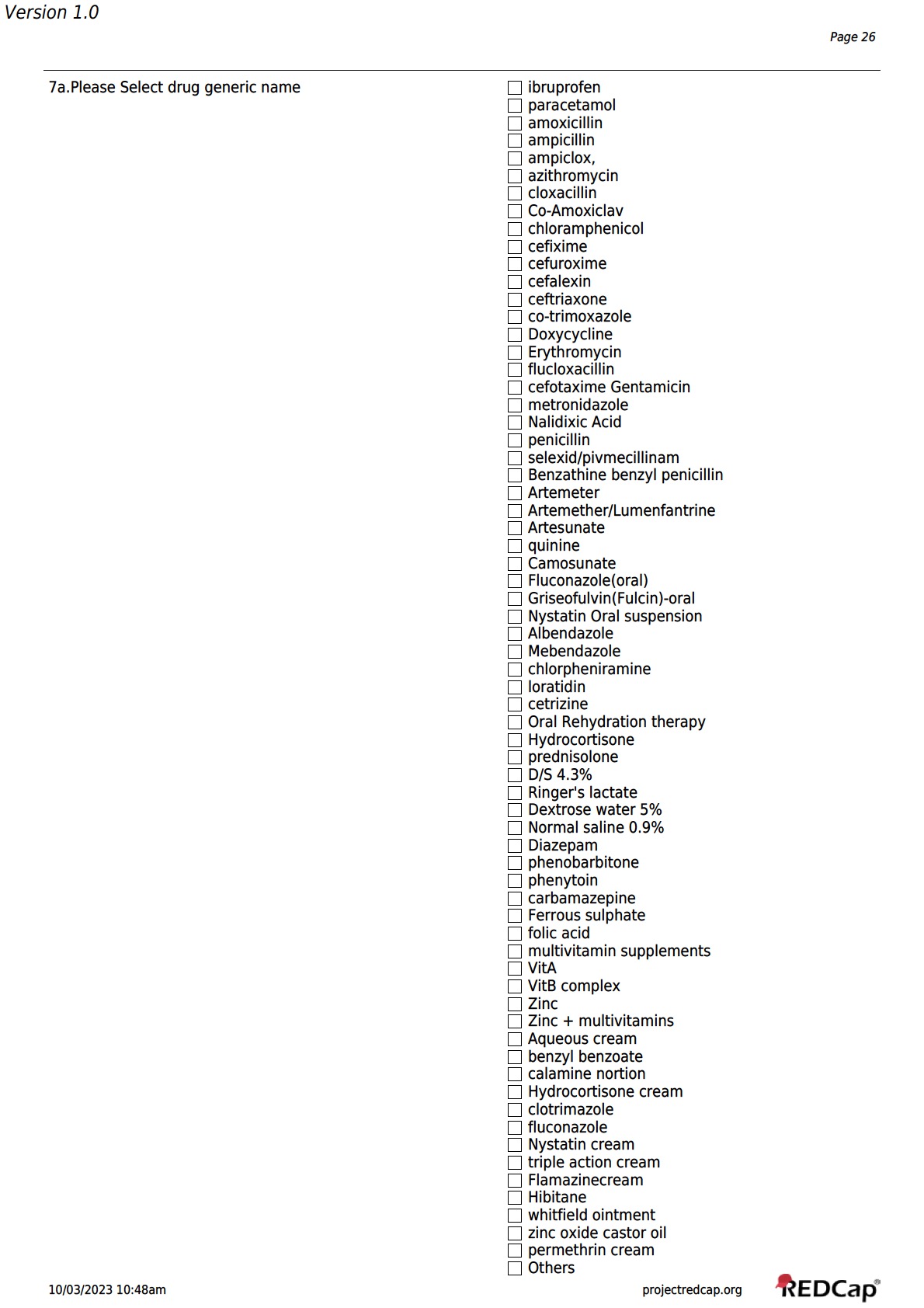

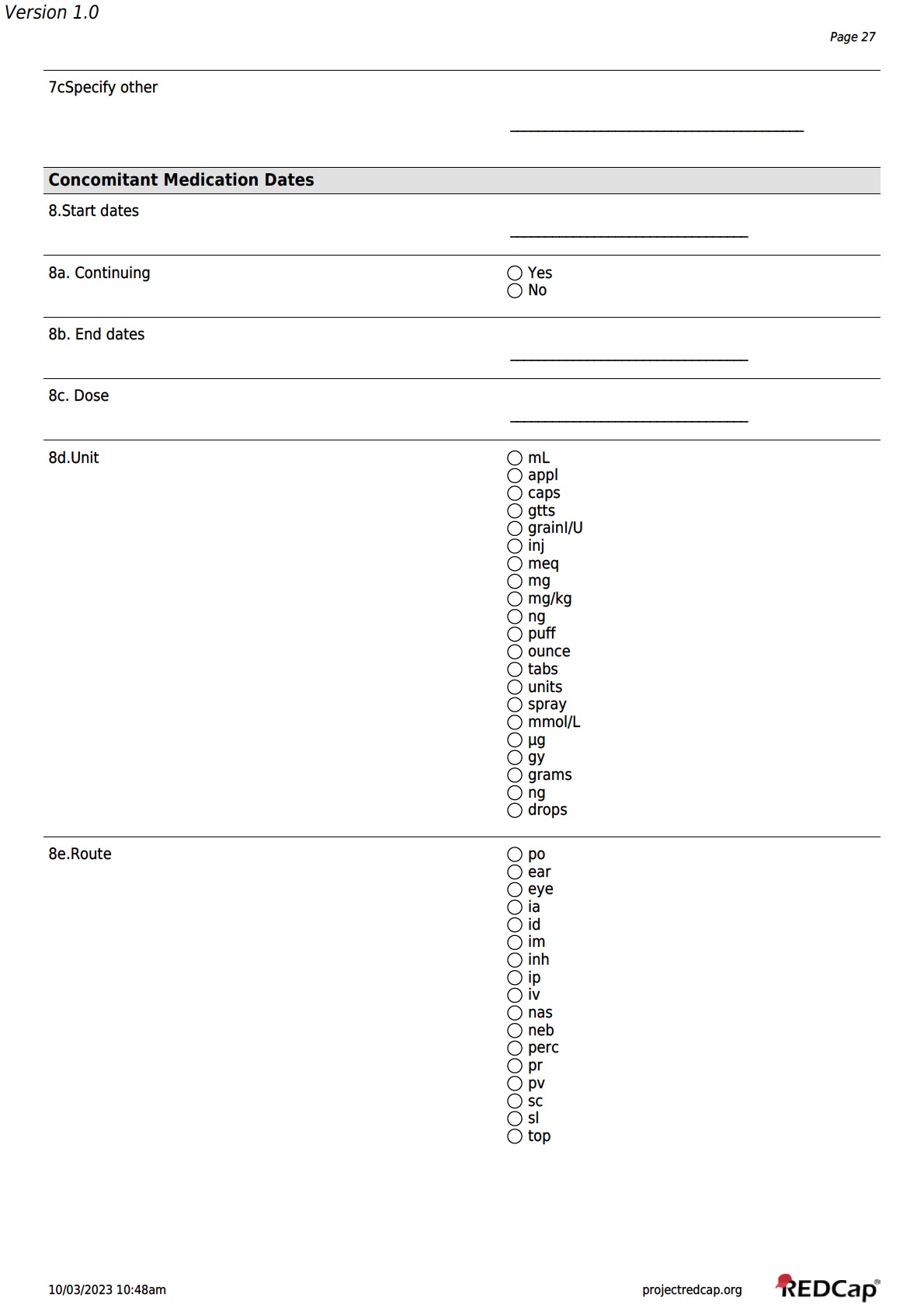

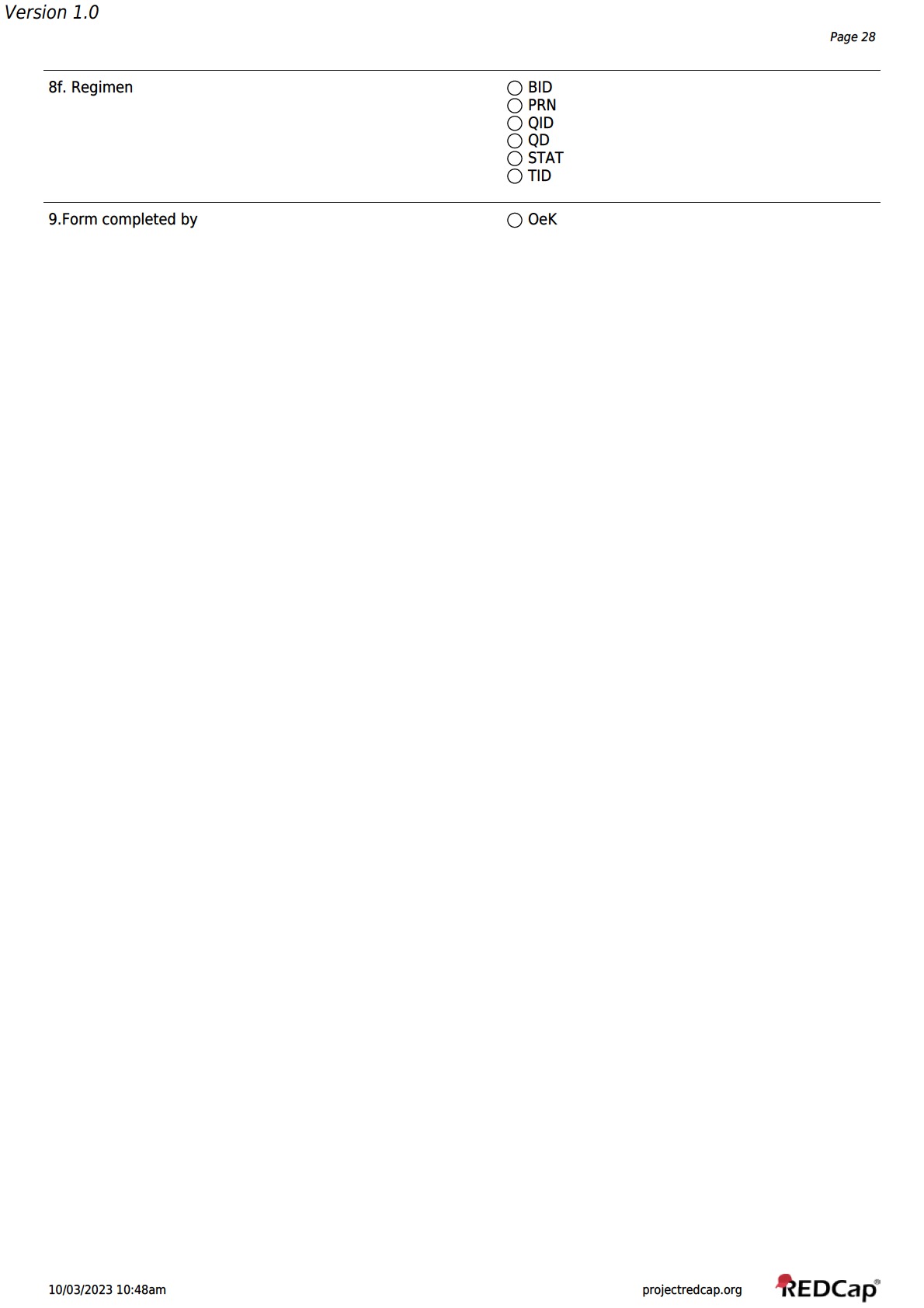

Supplement: Supplementary file 4 — Supplementary Material 4. [file 13063_2024_8101_MOESM4_ESM.docx]
